# Silencing of a Germin-Like Protein Gene (CchGLP) in Geminivirus-Resistant Pepper (*Capsicum* *chinense* Jacq.) BG-3821 Increases Susceptibility to Single and Mixed Infections by Geminiviruses PHYVV and PepGMV

**DOI:** 10.3390/v7122930

**Published:** 2015-11-25

**Authors:** Laura Mejía-Teniente, Ahuizolt de Jesús Joaquin-Ramos, Irineo Torres-Pacheco, Rafael F. Rivera-Bustamante, Lorenzo Guevara-Olvera, Enrique Rico-García, Ramon G. Guevara-Gonzalez

**Affiliations:** 1 C.A. Ingeniería de Biosistemas, Facultad de Ingeniería-Campus Amazcala, Carretera a Chichimequillas, Km. 1, S/N, El Marques, Queretaro C.P. 76229, Mexico; lauralyo@yahoo.com.mx (L.M.-T.); torresirineo@gmail.com (I.T.-P.); ricog@uaq.mx (E.R.-G.); 2 Instituto Tecnológico de Roque, Departamento de Ingeniería en Industrias Alimentarias, Km. 8 Carr. Celaya-J. Rosas, Roque, Celaya, Gto C.P. 38110, Mexico; ahuitzolt_jjr@yahoo.com.mx; 3 Departamento de Ingeniería Genética, Centro de Investigación y de Estudios Avanzados (CINVESTAV)-Unidad Irapuato, Carretera Irapuato-Leon, Km 9.6, Libramiento norte, Irapuato, Guanajuato A.P. 629, Mexico; rrivera@ira.cinvestav.mx; 4 Departamento de Ingeniería Bioquímica, Instituto Tecnológico de Celaya, Ave. Tecnológico y A, Garcia-Cubas, S/N, Col. FOVISSSTE, Celaya, Gto A.P. 57, Mexico; lorenzogo@yahoo.com

**Keywords:** VIGS, plant-virus interaction, resistance, Mn-SOD, transient expression, Germin-like proteins, J0101

## Abstract

Germin-like proteins (GLPs) are encoded by a family of genes found in all plants, and in terms of function, the GLPs are implicated in the response of plants to biotic and abiotic stresses. CchGLP is a gene encoding a GLP identified in a geminivirus-resistant *Capsicum chinense* Jacq accession named BG-3821, and it is important in geminivirus resistance when transferred to susceptible tobacco in transgenic experiments. To characterize the role of this GLP in geminivirus resistance in the original accession from which this gene was identified, this work aimed at demonstrating the possible role of CchGLP in resistance to geminiviruses in *Capsicum chinense* Jacq. BG-3821. Virus-induced gene silencing studies using a geminiviral vector based in PHYVV component A, displaying that silencing of CchGLP in accession BG-3821, increased susceptibility to geminivirus single and mixed infections. These results suggested that CchGLP is an important factor for geminivirus resistance in *C. chinense* BG-3821 accession.

## 1. Introduction

Geminiviridae is a plant virus family whose members infect important crops worldwide. These viruses contain single-stranded DNA genomes packed in twinned particles [1]. Family Geminiviridae comprises genera: Mastrevirus, Begomovirus, Curtovirus, Topocuvirus, Becurtovirus, Eragrovirus and Turncurtovirus [2,3]. Genus Begomovirus is the most widespread and diverse worldwide, causing crop losses ranging from 30% to 100% [4]. Pepper Huasteco Yellow Vein Virus (PHYVV) and Pepper Golden Mosaic Virus (PepGMV), are Begomoviruses widely distributed in Mexico, and considered major viral pathogens in pepper [5,6,7]. Accession BG-3821 of *Capsicum chinense* Jacq. was identified and characterized as a source of resistance to PHYVV and PepGMV [8,9,10,11,12,13]. Transcriptomic studies with accession BG-3821, identified a germin-like protein (GLP) gene called CchGLP, as involved in resistance to PHYVV and PepGMV [14]. CchGLP displayed a manganese-superoxide dismutase (Mn-SOD) in bacterial heterologous expression systems [15]. Transgenic expression of CchGLP in a geminivirus-susceptible tobacco cultivar (Nicotiana tabacum xanthi nc) provided resistance to geminivirus infections [7]. GLPs have been classified based on oxalate oxidase (OXO) or superoxide dismutase (SOD) activities [16]. GLP activity results in production of hydrogen peroxide, a reactive oxygen species (ROS) important in plant signaling to stress. Hydrogen peroxide is a signal for hypersensitive cell death [17,18]. Leaves of *C. chinense* accession BG-3821 displayed higher accumulation of ROS in comparison to susceptible accessions [13]. Virus-induced gene silencing (VIGS) is a virus vector technology that exploits RNA-mediated defense mechanisms in plants to specifically silence genes of interest [19]. Once plant cell become virus-infected, small interfering RNA (siRNA) is produced in the infected cell corresponding to parts of the viral vector genome, including any non-viral insert. VIGS technology is a powerful approach to functional genomics and to knock out function of a gene-of-interest [20]. The first RNA virus used as a silencing vector was Tobacco mosaic virus (TMV) [21]. Tobacco rattle virus (TRV) vectors have also been used in successful silencing studies of PDS and FLO genes (Phytoene desaturate and Floricaula genes respectively) in Cysticapnos vesicaria causing strong photobleaching of green parts and reduction of endogenous PDS transcript levels and affected floral phyllotaxis, symmetry and floral organ identities [22]. Infection with an apple latent spherical virus (ALSV) vector containing a fragment of soybean isoflavone synthase 2 (soyIFS2) gene reduced the levels of both soyIFS2- and soyIFS1-mRNAs and then the isoflavone content in cotyledons of mature seeds of infected soybean plants [23]. Rice tungro bacilliform virus (RTBV) has been developed as a rice-infecting virus containing DNA as the genetic material. Agroinoculation mediated transfection accumulated a full-length RTBV DNA cloned as a partial dimer in a binary plasmid and produced detectable RTBV coat proteins within two weeks of infection [24]. For VIGS studies in plants, geminiviruses present several advantages as their genome organization is conserved, so new geminiviruses can be easily exploited using basic knowledge available of these viruses [25]. The small genome of geminiviruses is very convenient for cloning and sequencing, and its propagation is simpler than the *in vitro* production of RNA. In addition, DNA is less labile than RNA, and can be directly inoculated, avoiding the need to use Agrobacterium as a carrier. Furthermore, DNA vectors have proven to be more stable than RNA vectors because they lack an RNA intermediate and, thus, are not targets for RNA degradation. Hence, even if their transcripts are silenced, the replicons continue to multiply and move, although at a slower rate than their wild type equivalents [20]. By contrast, RNA viruses induce a transient silencing that might result in the removal of the viral genome itself or in important differences in transcripts levels [26]. In this sense, a Pepper huasteco yellow vein virus (PHYVV-A)-derived vector, has been evaluated for silencing genes Comt, pAmt, and Kas in pepper plants which are involved in the biosynthesis of capsaicinoids. This approach was successful in silencing the aforementioned genes and leading to a dramatic reduction in capsaicinoid content [27]. Thus, the aim of this work was to evaluate the role of CchGLP in natural resistance to geminivirus in *Capsicum chinense* BG-3821 through silencing the gene using PHYVV-A derived vector.

## 2. Materials and Methods

### 2.1. Plant Growth

*Capsicum chinense* BG-3821 plants, previously characterized as resistant to geminivirus [8,9,10], were employed for testing silencing of CchGLP. Seeds (supplied by CINVESTAV-Unidad Irapuato) were germinated in plastic pots (250 cm^3^) containing a sterile substrate (peat moss:vermiculite in 3:1 proportion), and grown in a chamber at 28 °C under a photoperiod of 16/8 h light/dark.

### 2.2. Construction of Viral Vector for Silencing CchGLP

**Figure 1:**
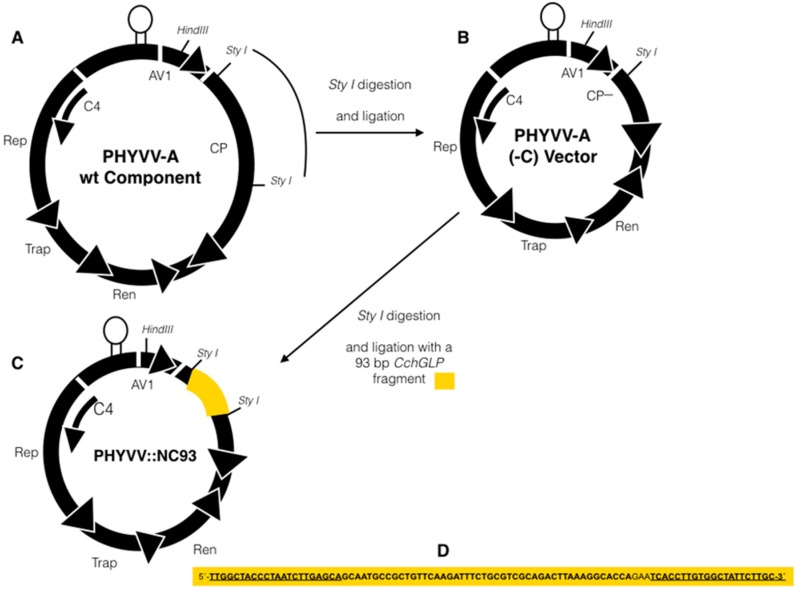
Construct design of PHYVV::NC93 (a PHYVV-A derived vector) for CchGLP silencing in *C. chinense* BG-3821 plants. (**A**) wild type (wt) PHYVV component A; (**B**) PHYVV-A (-C) vector resulted from Sty I digestion of wt PHYVV component A; (**C**) PHYVV::NC93 silencing vector carrying a 93 base pairs (bp) fragment of the 5’ region of CchGLP ORF (section in yellow); (**D**) DNA sequence of the 93 bp from CchGLP ORF used for silencing studies. Underlined are indicated the sequences used for primers (in each primer a Sty I site was included at the 5′ region of each primer, see materials and methods). PHYVV-A component is cloned in the Hind III of plasmid bluescript SK+.

PHYVV-A (-C) vector cloned into the Hind III site of plasmid bluescript SK+ was kindly provided by Dr. Rafael Rivera-Bustamante from CINVESTAV Unidad Irapuato, and previously evaluated for functional silencing in pepper plants [27]. To construct the silencing vector for CchGLP, a fragment of 93 bp by PCR from 5′-end of CchGLP open reading frame (ORF) was produced, which did not belong to the cupin domain of the GLP, using initiators that contain the recognition sequence to the restriction enzyme Sty I, because this enzyme is within the coat protein (CP) gene of the viral vector PHYVV-A (-C). For this purpose, PCR oligonucleotides were designed from CchGLP cDNA (accession number DQ677335): 5′-ccttggTTGGCTACCCTAATCTTGAGCA-3′ (forward primer) and 5′ccaaggGCAAGAATAGCCACAAGGTGA-3′ with the following amplification conditions: 94 °C/1 min, 63 °C/1 min and 72 °C/1 min by 35 cycles. Once the fragment of 93 bp was flanked by enzyme Sty I, single bands of the expected size were cloned directly into the pCR^®^ 2.1-TOPO^®^ TA vector—subcloning vector (Invitrogen, Carlsbad, CA, USA, https://www.lifetechnologies.com [28]) as indicated by the manufacturer protocol. Ligation tested in subcloning vector, the 93 bp fragment was cleaved with the restriction enzyme Sty I and this fragment was linked into the Sty I site of the vector viral PHYVV-A (-C), resulting in the PHYVV::NC93 (see Figure 1).

### 2.3. Inoculation of C. chinense BG-3821 Plants with Silencing Vector and Geminivirus Infections

Inoculation of *C. chinense* BG-3821 with the silencing vector and the geminiviruses was carried out in 4–6 true leaves stage using high pressure biolistic according to [8]. The inoculation of plants was developed in two steps: (a) First, plants were inoculated with silencing vector (PHYVV-A (93 bp CchGLP) + wt PHYVV-B9; (b) then, 5 days after this inoculation, the same plants were inoculated with either wt components A and B of PHYVV and/or PepGMV. Control plants, were inoculated with empty vector PHYVV-A (-C) plus PHYVV-B and in some cases mock-inoculated only with plasmid blue script SK+. Before inoculation, viral genomes were excised from the plasmid vector before inoculation using HindIII or BamHI digestions for PHYVV or PepGMV, respectively [8]). Inoculated plants were then incubated under the same conditions used in germination. Moreover, a replica for each treatment using a manual low pressure biolistic gun was carried out in order to analyze the systemic silencing. For this latter studies, plants were only inoculated with full (93 bp CchGLP) or empty (-C) vector without wt PHYVV-B. All biolistic inoculations were essentially carried out according to [8]. The experimental unit size for each treatment was 30 plants; experiments were carried out by triplicate in independent assays.

### 2.4. Detection of Geminiviruses by PCR

Total DNA extractions of the inoculated plants were carried out at 90 dpi with geminiviruses according to the modified method of Dellaporta [29]. The DNA obtained was used as a template in the PCR in order to analyze the movement of virus in inoculated plants. Specific oligonucleotides were used for each geminivirus: 240-5′-GGCTTATTTGTAATAAGAG-3′ and 241-5′-GAATTAAAGGTACATGGAC-3′ to PHYVV, and JM23-5′-TGGTGTAGGACTCCAGCAGAGTC-3′ and JM24-5′-TAGGCCCACACCTTGGTGACCAA-3′ to PepGMV. These oligonucleotides flank the common region of PHYVV and PepGMV and amplify a fragment of 350 and 280 bp, respectively. The conditions used for both oligonucleotides were: 95 °C/1 min, 53 °C/1 min and 72 °C/1 min by 35 cycles [5].

### 2.5. Assessment CchGLP Gene Silencing in C. chinense BG-3821

The silencing assessment of the CchGLP gene was conducted in two phenological stages (30 and 90 dpi). The expression of CchGLP gene was assayed at 30 dpi and 90 dpi by means of the reverse transcriptase-mediated polymerase chain reaction (RT-PCR) using 1 μg total RNA from foliar tissue. Then, total RNA of silenced-and-infected, and control (non-silenced) plants were obtained using the TRIZOL^®^ Reagent (Invitrogen), according to the manufacturer’s instructions. RNA integrity was verified by 1.2% agarose gel electrophoresis. The quantification and purity of total RNA were measured spectro-photometrically by relation of absorbance (260/280 nm). Later, the first cDNA chain of CchGLP of each plant sample was obtained by RT-PCR according to the manufacturer’s instructions (Superscript One-Step RT-PCR System; Invitrogen). Then the cDNA of CchGLP was amplified with specific oligonucleotides: 5′-TCTAGAATGTCTAAACTTATAATCT-3′, and 5′-GAGCTCCTAACCCTTGAGTTTCTT-3′ as described by [7] Guevara-Olvera *et al.*, that gave rise to a fragment of 612 bp, employing the following conditions: 94 °C/5 min; followed by 30 cycles of 94 °C/1 min, 60 °C/1 min; finally 72 °C for 7 min.

### 2.6. Evaluation of the Phenotypic Response

The phenotypic response was analyzed daily once biolistic inoculation was carried out. Results were registered and reported at 30 and 90 dpi. The scale of severity for geminivirus disease used was according to [5,8].

### 2.7. Statistical Analysis

Data were subjected to analysis of variance by the general linear models (GLM) procedure, means comparison by Tukey’s test according to SAS methods [30].

## 3. Results

### 3.1. Phenotypic Response in C. chinense BG-3821 CchGLP-Silenced Plants

Figure 1 shows the PHYVV-A based vector used to silence CchGLP in *C. chinense* Jacq. BG-3821. A 93 base pairs (bp) 5’ region of CchGLP ORF, was inserted into the Sty I sites within CP ORF of PHYVV-A. This 93 bp region does not include homologous sections with other GLPs reported elsewhere, including one identified in the transcriptomic studies where CchGLP was originally observed (not shown). This latter consideration is important in order to specifically silence CchGLP with no other GLPs present in accession BG-3821. It is worth mentioning that fragments of 103 and 535 bp of CchGLP were also evaluated with similar results, however the 93 bp fragment displayed more stable results in this study, thus this fragment was chosen for CchGLP silencing studies reported in this work (data not shown).

**Figure 2:**
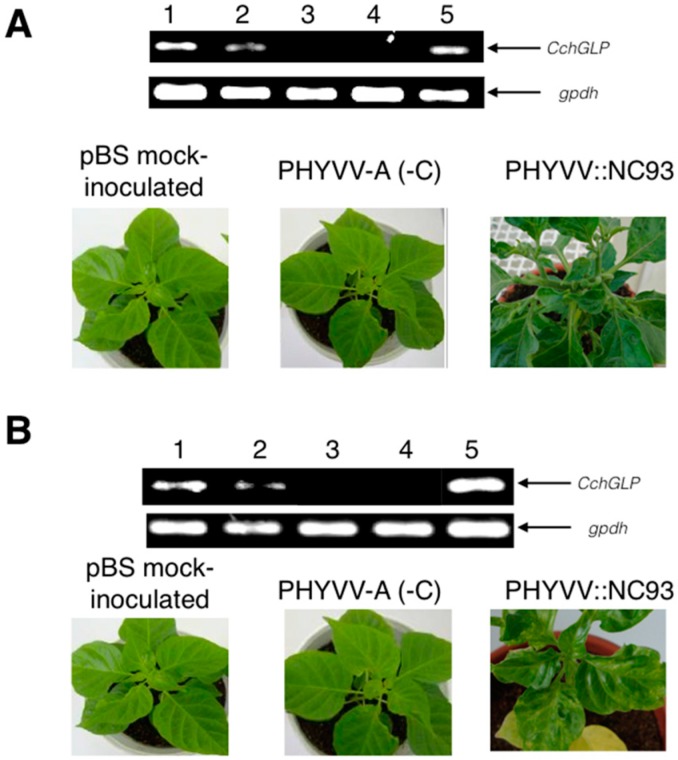
Molecular and phenotypical analysis in *C. chinense* BG-3821 using PHYVV::NC93 construction. (**A**) lane 1, pBS mock-inoculation; lane 2, PHYVV-A (-C); lane 3, PHYVV::NC93 silenced plant; lane 4, − control; lane 5, + control; (**B**) lanes 1–5 corresponds to the same treatments as in panel A. In panels A and B, the bottom section displays symptomatology at 30 days post-inoculation (dpi) of either PHYVV (panel A) or PepGMV (panel B) wt components A and B, respectively. Negative control corresponds to plants pBS mock-inoculated. Positive control samples came from plants sprayed with salicylic acid 0.01 mM, which is a potent CchGLP inductor as reported by [14]. A housekeeping gene (gpdh, encoding glyceraldehyde phosphate dehydrogenase) was used as constitutive control.

**Figure 3:**
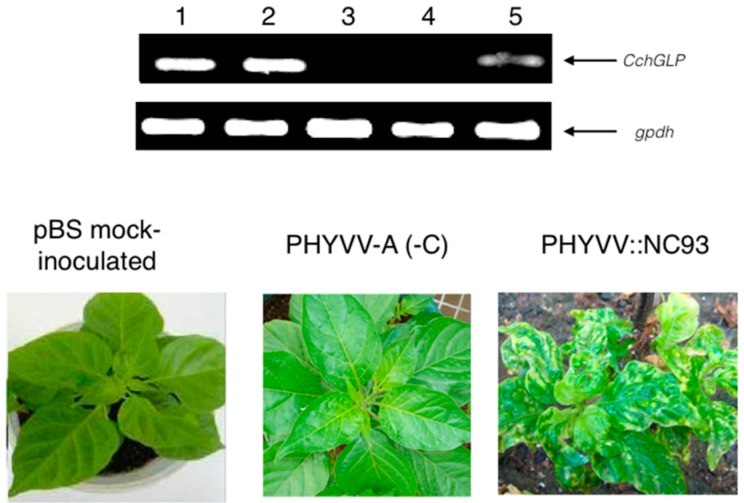
Molecular and phenotypical analysis in *C. chinense* BG-3821 using PHYVV::NC93 construction. Lane **1**, pBS mock-inoculation; lane **2**, PHYVV-A (-C); lane **3**, PHYVV::NC93 silenced plant; lane **4**, − control; lane **5**, + control. Panel B, lanes 1–5 corresponds to the same treatments as in panel A. In the bottom section symptomatology is displayed at 30 days post-inoculation of mixed infection by both PHYVV and PepGMV wt components A and B. Negative control corresponds to plants pBS mock-inoculated. Positive control samples came from plants sprayed with salycilic acid 0.01 mM, which is a potent CchGLP inductor as reported by [14]. A housekeeping gene (gpdh, encoding glyceraldehyde phosphate dehydrogenase) was used as constitutive control.

Figure 2 and Figure 3 show the molecular and phenotypic analysis of typical CchGLP-silenced plants with viral vector system based on PHYVV-A, once single inoculated either with PHYVV (Figure 2A), PepGMV (Figure 2B), or mixed inoculated (Figure 3). Control plants inoculated with empty viral vector (PHYVV (-C) + wt PHYVV component B), displayed no symptoms. Plants were evaluated 30 days post-geminivirus inoculation (dpi). It is clearly shown that when CchGLP is silenced, (Figure 2A,B), the phenotypic response in accession BG-3821 is the appearance of symptoms in comparison to non-silenced control plants.

**Figure 4:**
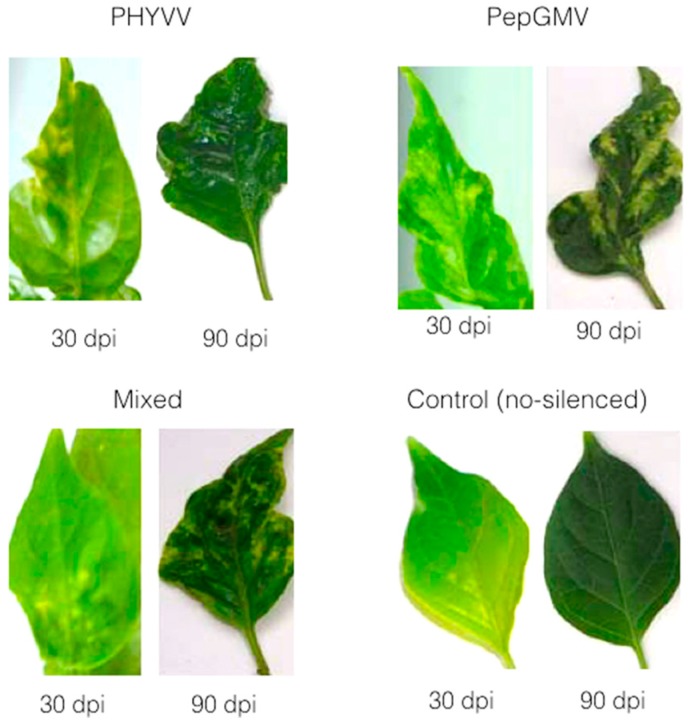
Typical symptoms in upper leaves of CchGLP-silenced *C. chinense* BG-3821 at 30 and 90 dpi with PHYVV, PePGMV and mixed infections. Control means plants inoculated only with empty PHYVV-A vector + wt component B and then PHYVV and PepGMV mixed infected.

It is important to mention that in this work, 80% of inoculated-plants with silencing vector displayed no CchGLP expression throughout the plant (indicating successful silencing; data not shown). Thus, results in Figure 2 and Figure 3 correspond to these silenced plants, in order to evaluate further their phenotype. In all cases CchGLP expression was silenced, plants became susceptible to geminivirus single and mixed infections (Figure 2 and Figure 3). Typical symptomatology in upper leaves at 30 and 90 days post-geminivirus inoculation in CchGLP-silenced plants is shown in Figure 4. Symptoms increased in severity from 30 to 90 dpi both in single and mixed infections. Control plants (inoculated with empty PHYVV-A vector, and then infected with wild type geminivirus), did not show any symptoms in these experiments (Figure 4).

This latter result indicated that phenotypic response of BG-3821 CchGLP-silenced plants increased symptomatology when geminivirus-infected. These results suggested that CchGLP was still silenced at 90 dpi, and support the hypothesis of the role of CchGLP as an important factor for geminivirus-resistance in *C. chinense* BG-3821.

### 3.2. Analysis of Systemic Silencing of CchGLP

**Figure 5:**
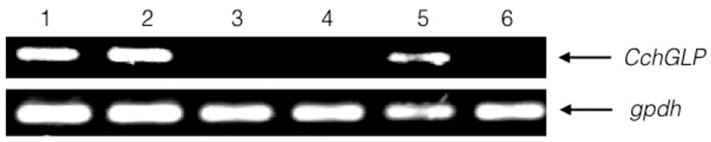
Silencing of CchGLP at 90 dpi in originally inoculated and non-inoculated leaves in *C. chinense* BG-3821 plants. Lanes **1** and **2**, upper and lower leaves in inoculations with empty viral vector, respectively; Lanes **3** and **4** upper and lower leaves of plants inoculated with silenced vector for CchGLP, respectively; Lane **5**, + control (geminivirus mixed infected BG-3821 plant); lane **6** − control (inoculated plant only with empty vector). The housekeeping gene gpdh (encoding glyceraldehyde phosphate dehydrogenase) was used as control.

The systemic silencing verified in plants inoculated by low pressure biolistic due to the shot is specifically directed to a specific leaf in the plant. Thus, upper and lower non-inoculated leaves were analyzed for CchGLP expression. Figure 5 displays the results of CchGLP expression in upper and lower leaves. As shown, both in upper and lower leaves CchGLP expression was detected in the cases of expected gene expression (Figure 5, lanes 1, 2, and 5). In this sense, CchGLP silencing at 90 dpi was still present after the full-silencing vector was inoculated (Figure 5, lanes 3 and 4). It is worth mentioning that 95% of CchGLP-silenced and then becoming geminivirus-susceptible plants, kept the susceptible phenotype at least during 90 dpi in both single and mixed infections. Thus, the systemic silencing of CchGLP even at 90 dpi suggests that in these cases this could be the explanation of the phenotype. However, the rest 5% of these originally-silenced and geminivirus-susceptible plants showed a slight expression of the CchGLP gene, and came to display slight symptoms in newly developed leaves, corresponding with a glitch in CchGLP-silencing in these plants (not shown).

**Figure 6:**
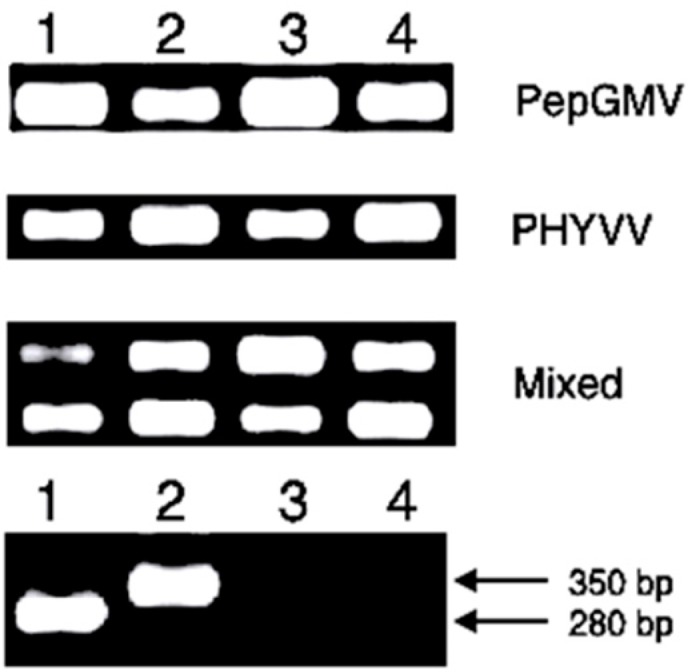
Detection by PCR of common region of PHYVV and PepGMV at 90 dpi in lower and upper leaves in *C. chinense* BG-3821 either in single or mixed infections. Lanes **1** and **3**, lower and upper leaves in silenced plants, respectively; Lanes **2** and **4**, lower and upper leaves in no-silenced plants. In the bottom section, lane 1, positive control of wt PepGMV component A (280 bp); lane 2, PHYVV Component A (350 bp); Lane 3, negative control, of DNA from a pBS mock-inoculated plant; lane 4, negative control of un-inoculated plant.

To verify the presence of geminiviruses throughout the evaluated plants, detection studies of geminiviral DNA in apical and basal leaves were carried out (Figure 6). These results indicated the movement and replication of these viruses either in single or mixed infected plants. These results agree with those reported elsewhere [8,9].

### 3.3. Severity of Disease Analysis

The severity of disease caused by geminiviruses in silenced and non-silenced CchGLP plants was evaluated (Table 1). In general it was shown that CchGLP-silenced plants displayed a significant increase in severity in comparison to non-silenced plants. However, when compared to susceptible accessions of *C. chinense* (UX-SMH 1), the severity levels were milder, suggesting that CchGLP is not the only factor determining geminivirus resistance in *C. chinense* Jacq. BG-3821 (Table 1).

**Table 1:** Severity * of disease in silenced and non-silenced *C. chinense* BG-3821 plants.

Treatment	Severity in CchGLP Silenced *C. chinense* BG-3821	Severity in CchGLP Non-Silenced *C. chinense* BG-3821 Plants	Severity in Susceptible *C. chinense* Plants (Accession UX-SMH-1)
	30 dpi 90 dpi	30 dpi 90 dpi	30 dpi 90 dpi
PHYVV	3b 5b	0a 0b	5c 8b
PepGMV	3b 6a	0a 1a	7a 9a
Mixed: PHYVV + PepGMV	4a 6a	0a 1a	6b 9a
Mock-inoculated (empty viral vector + wt PHYVV component B)	0c 0c	0a 0b	0d 0c

* Severity is according to a scale reported [5,8]. Data shown are the average of 60 plants analyzed in three independent experiments. Different letters in each column for each dpi designates a significant difference (Tukey *p* < 0.05).

## 4. Discussion

In summary, the aforementioned results suggested that the GLP protein encoded by CchGLP of *C. chinense* BG-3821 could be actively participating in the defense mechanism of these plants due to in non-silenced plants inoculated with the empty vector (PHYVV (-C) + wt component B), resistance was displayed to simple and mixed infection with geminiviruses, and this phenotype became susceptible when silencing CchGLP. Moreover, in [7] we showed that CchGLP gene supported the resistance, either by attenuation and delay of symptoms, in single and mixed geminivirus infections in geminivirus-susceptible Nicotiana tabacum xanthi nc transgenic plants.Previous studies have shown the high expression of CchGLP gene in plant-geminivirus interaction in *C. chinense* BG-3821, as well as using salicylic acid (SA) as an inductor [12,14,15]. Park *et al.* [31] showed a similar pattern of expression of CaGLP1 in inoculated plants with TMV-Po virus, and they suggested that the transcript of CaGLP1 gene could be involved in the defense response of plants, which was caused by the compatible interaction between resistant plants and avirulent pathogens. Additionally, they [31] observed a rapid accumulation of CaGLP1 transcripts by SA 6 h after the induction. In the case of accession BG-3821, at least 2 h after the induction with SA, the expression of CchGLP gene was detected [12]. According to the results obtained either at 30 and 90 dpi, the efficiency of silencing was kept to the flowering stage in most of the silenced plants (95%). The lack of expression of CchGLP after the silencing and the phenotypic events of susceptibility generated by viral infection in silenced plants suggested that CchGLP could be part of the resistance mechanism or one of the main elements of this mechanism against geminiviruses in accession BG-3821. A better explanation for this event of susceptibility might be related to the levels of H_2_O_2_ in cells. According to Lou *et al.* [32,33], the silencing of a GLP from Nicotiana attenuata significantly decreased the H_2_O_2_ production. These authors demonstrated that H_2_O_2_ induction by oral secretion of the mean depredator of Nicotiana attenuata, Manduca sexta protected the plants against the pest; however, silencing a GLP in Nicotiana attenuata stimulated its depredator activity [29]. H_2_O_2_ is a molecule that is generated by SOD activities during an oxidative burst and is important in signaling against stress in plants [18]. H_2_O_2_ signaling pathway reacts to different stimuli, such as biotic and abiotic, plant hormones (*i.e.*, SA) and, to processes of cell division and cell growth [33]. Furthermore, it can activate defense genes involved in the production of the biochemical arsenal of defense in the plants against stress. We analyzed the resistance to geminivirus in transgenic plants of Nicotiana tabacum xanthi nc expressing CchGLP [7], which is highly susceptible to PHYVV and PepGMV infections. The results showed that transgenic lines of N. tabacum xanthi nc presented delay and amelioration of symptoms while the non-transgenic control was highly susceptible and its production of H_2_O_2_ was lower than in transgenic lines.Together these results suggested that in absence of CchGLP expression, the Mn-SOD activities associated is null, causing a decrement of H_2_O_2_ levels in cells. Therefore, the lack of CchGLP expression might decrease the response signals to geminivirus in *Capsicum chinense* BG-3821. Park *et al.* [31] observed a similar response generated by CaGLP1 gene, which was induced by TMV-Po virus, and they located the CaGLP1 transcripts in the cellular surface of *Capsicum annuum*. Likewise, it is suggested that CchGLP accompanies stress response and is associated with the development of the general mechanism of defense against pathogens.

## 5. Conclusions

Taken together these results, it is concluded that CchGLP is an important component in the resistance to geminiviruses in *C. chinense* accession BG-3821.
